# Fulton County (Ill’s.) Medical Society

**Published:** 1851-07

**Authors:** G. H. Hickman, W. M. McDowell

**Affiliations:** President; Sec’y.


### ARTICLE VI.

### *Fulton County (Ill's.) Medical Society.*

Agreeably to adjournment, the Fulton County Medical Society convened at Mr. Maple's Hall, in Canton, on Saturday the 24th of May, G. H. Hickman being in the Chair, and W. H. McDowell acting as Secretary.

The object of the meeting having been stated, the following resolution was adopted:

Whereas, we cannot recognize Dr. Falkenthal as a regular practitioner of medicine, nor as eligible to membership of this Society; therefore,

Resolved, That he be suspended as a member of the committee appointed to draft and report a Constitution and By-Laws, and that Dr. Wm. H. Nance, be substituted a member of said committee.

The committee previously appointed to draft a Constitution, reported.

On motin the report was accepted and the committee discharged.

On motion it was resolved that a committee be appointed to draft a bill of rates; whereupon Messrs. E. C. Gardiner, J. H. Pearsoll, J. Gregory, and J. R. Walters were appointed said committee.

On motion the society adjourned until one o'clock P. M.

Met pursuant to adjournment. A Constitution and By-Laws were then adopted. The report of the committee on the bill of rates was read, and on motion was laid on the table.

On motion it was resolved to proceed to the election of officers for the ensuing year; which resulted in the election of

W. H. Nance, President; G. H. Hickman, A. Hull, and J. B. E. Albright, Vice-Presidents; Asa Lee Davidson, Recording Secretary; E. C. Gardiner, Corresponding Secretary; J. R. Walters, Treasurer; H. Martin, Librarian; H. Ingersoll, A. M. Johnston, J. C. Fitz, J. H. Pearsoll and J. Gregory, Censors.

The delegates appointed to the State Convention were G. H. Hickman, J. B. E. Albright, J. C. Fitz, and A. Hull.

On Motion, Resolved, That if any of the delegates, appointed to the State Convention, were absent, their vacancies be filled by visiting members.

On motion, Resolved, That the proceedings of this meeting be published in the Canton Register, and the North-Western Med. and Surg. Journal.

On motion, Resolved, That we tender our thanks to Mr. Maple, for the use of his hall.

Physicians present were G. H. Hickman, W. H. Nance, J. C. Fitz, A. M. Johnston, E. C. Gardiner, J. R. Urich, Asa Lee Davidson, H. Ingersoll, J. R. Walters, H. Martin, J. B. E. Albright, W. M. McDowell, J. Gregory, A. Hull, W. D. Nelson, J. H. Pearsoll, and J. B. Cunningham.

On motion the meeting adjourned.

G. H. HICKMAN, President.

W. M. McDowell, Sec'y.

